# Combined anatomical reduction plate for quadrilateral acetabular fractures via a posterior approach: an anatomical–morphological study

**DOI:** 10.1186/s12891-024-07522-x

**Published:** 2024-05-28

**Authors:** Bao Chongshuai, Yan Xuhang, He Li, Yao Qingshuang, Chen Lin, Ao Jun

**Affiliations:** Department of Orthopaedic Surgery, The Affiliated Hospital of Zunyi Medical University, Zunyi, Guizhou 563000 China

**Keywords:** Acetabular fracture, Quadrilateral region, Reduction and internal fixation, Digital measurement

## Abstract

**Objective:**

To digitally measure the fixation trajectory of anatomical plates used in the combined reduction of quadrilateral acetabular fractures via the posterior approach, and to develop anatomical plates that align with the characteristics of the pelvis in the Chinese population.

**Methods:**

Pelvic computed tomography (CT) data from 102 adult patients were collected at the Affiliated Hospital of Zunyi Medical University. This group included 51 males and 51 females, aged between 20 and 60 years. Using Mimics software (version 21.0), a three-dimensional model of each pelvic data point was reconstructed. The fixation path for the combined reset anatomical steel plate was drawn, where the curves on the fixation path were approximated as arcs. The radius of curvature and length of these curves were measured, and an anatomical steel plate was designed to best fit the pelvic structure.

**Results:**

The combined anatomical reduction plate fixation system for quadrilateral acetabular fractures using a posterior approach consisted of two parts: a locking plate and a reduction plate. The posterior wall region (r2), ischial region (r3), quadrilateral region (r4), and bending region (r5), and the total length of the reduction plate were significantly smaller in females (*P* < 0.05). Similarly, the posterior wall region (R3), distal posterior wall region (R4), and the total length of the locking plate were significantly smaller in females (*P* < 0.05). Additionally, the anterior superior iliac spine side (r1) and the total length of the T-shaped auxiliary plate were significantly smaller in females (*P* < 0.05). The bending angle (< A) was also significantly smaller in females (*P* < 0.05).

**Conclusions:**

The pelvic surface structure is irregular and varies greatly among individuals.Compared to the traditional steel plate, The combined reduction anatomical plate designed in this study demonstrated high precision and improved conformity to the anatomical structure of the pelvis.

## Introduction

Acetabular fractures are complex intra-articular fractures often accompanied by multiorgan dysfunction. Due to their deep anatomical location and intricate structure, the treatment of acetabular fractures remains a significant challenge for orthopedic surgeons. Key factors such as anatomical reduction of the fracture, rigid internal fixation, and early functional exercise are crucial for optimal outcomes [1–4]. . In recent years, an increase in traffic and construction accidents has led to a rise in acetabular fractures [5]. The standard treatment for unstable acetabular fractures involves open reduction and internal fixation, The prognosis of surgery is good and the incidence of complications is low [6–8]. 

Before the 1960s, conservative treatments like traction reduction and external orthosis were commonly employed for complex acetabular fractures affecting quadrilateral surfaces [9, 10]. However, with continuous medical advancements and a deeper understanding of anatomical structures, surgery have become the preferred method for treating these fractures. Traditional internal fixation options for displaced affecting quadrilateral surfaces fractures include wire-plate composite systems [11], T-plates [12], I-plates [13], iliosacral plates [14], and posterior column plates [15]. Although these surgical techniques and internal fixation devices have made some advances in the treatment of involved quadrilateral fractures, they still have some shortcomings, such as the requirement of high surgical skill and operator experience, a higher incidence of postoperative traumatic arthritis, the possibility of greater trauma, a prolonged operative time, increased intraoperative bleeding, and an increased probability of postoperative complications [13, 16, 17]. To overcome the shortcomings of traditional plates, domestic and foreign researchers have conducted numerous studies on pelvic rim screws [18], suprapubic quadrilateral body surface support plates [19], and a new dynamic anterior plate-screw fixation system; [20] however, considerable controversy remains regarding the optimal surgical treatment of quadrilateral fractures.

**Fig. 1:**
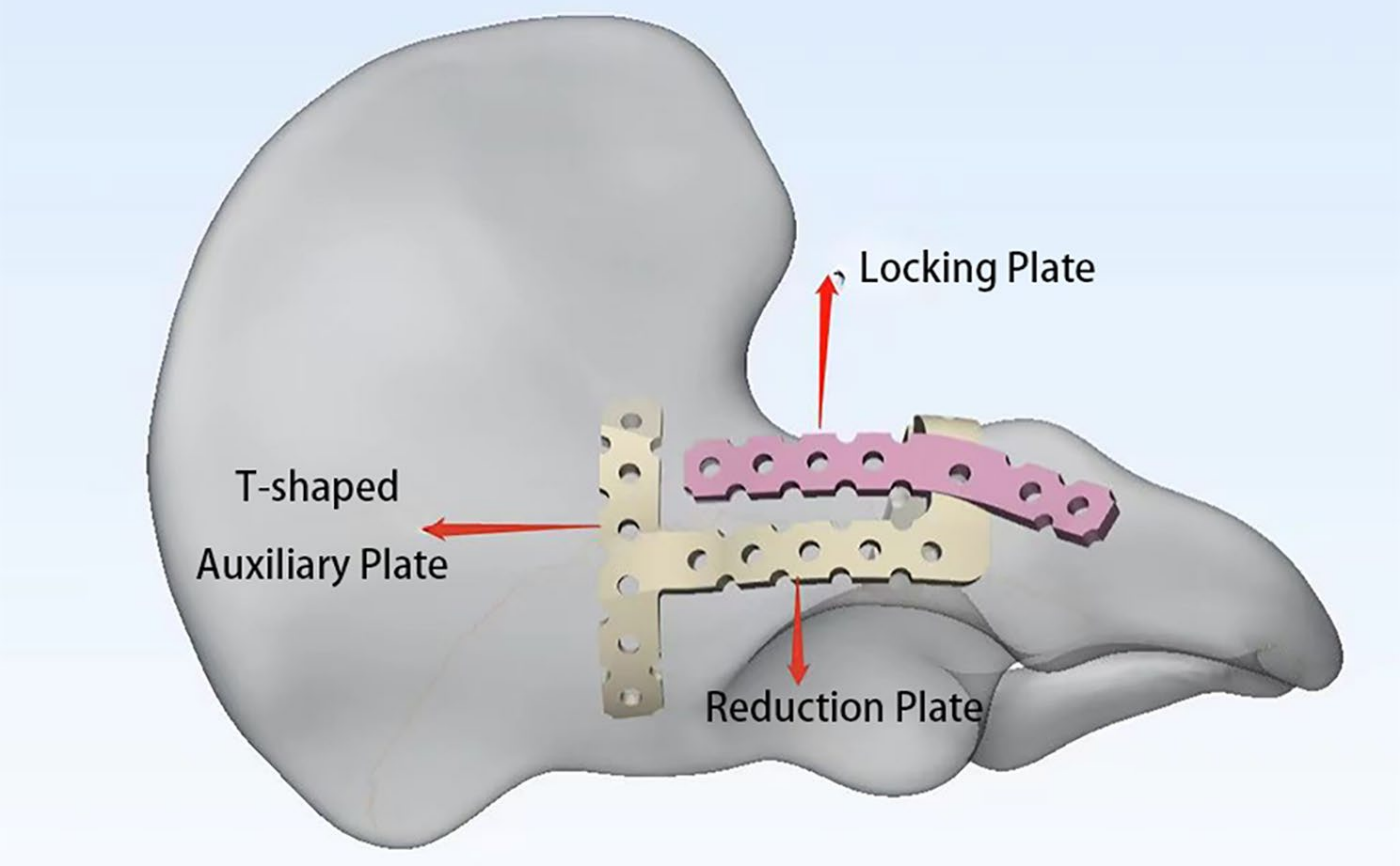
Combined reduction anatomical plates

Therefore, we aimed to innovatively design a novel shape for the reconstruction steel plate, named the combined reduction anatomical plate. This fixation system consists of two parts: a locking plate and a reduction plate. The locking plate is a curved strip plate that fits above the large sciatic notch. The reduction plate consists of the first plate, the second plate, the third plate, and the T-type auxiliary plate. The first plate is bent to fit the anatomical structure above the acetabulum, the second plate intersects with the locking plate, the third plate is bent to fit the anatomical structure of the acetabular quadrilateral body, and the T-type auxiliary plate is molded integrally with the first plate and fixed to the anterior column of the acetabulum (shown in Fig. 1). This fixation system has been granted a Chinese Patent (Patent No. CN202210974276.4).

However, this fixation system must be shaped based on the characteristics of the acetabulum and the surgeon’s experience. For inexperienced surgeons, poor precision of intraoperative plate shaping may lead to a loss of reset. To achieve a standard for the combined reduction anatomical steel plate with higher precision, a better fit to the pelvic surface anatomical structure, a reduction in operation time, and an improvement in therapeutic effect are required; therefore, this study utilized a three-dimensional (3D) model of the pelvis, measured the anatomical morphology parameters of the bone surface on the fixation path of this fixation system, and summarized the features of the plate to design and develop an anatomical steel plate with higher precision and in line with the pelvic structure of the Chinese population.

## Materials and methods

We collected data from adult patients who underwent pelvic CT + 3D at the affiliated hospital of Zuni Medical University between January 2022 and January 2023. A total of 102 cases of partial pelvic data were obtained, including 51 males with an age range of 22–60 years and an average age of 44.37 years, and 51 females with an age range of 23–60 years and an average age of 47.88 years. In this study, all patients were required to meet the following inclusion criteria: no lesions, fractures, tumors, or anatomical abnormalities on one or both sides of the pelvis and age between 20 and 60 years.

**Fig. 2:**
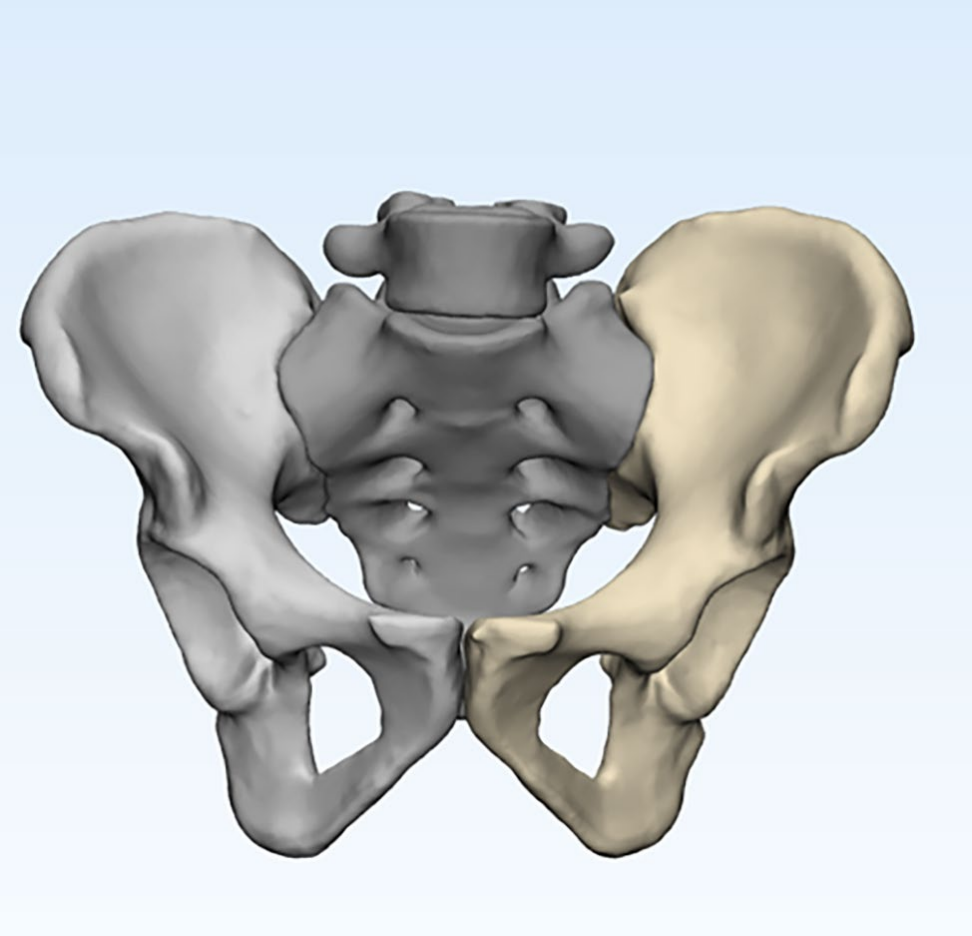
Three-dimensional model of the pelvis

All CT data were acquired using a 64-channel helical CT scanner with a slice thickness of 1 mm, and were saved in DICOM format. The DICOM CT data were imported into Mimics 21.0 (Materialise Company, Leuven, Belgium). Then, through threshold segmentation, region growing, and smoothing to remove soft tissue, a 3D pelvic model was reconstructed and saved in STL format. The STL-format pelvic 3D model was further processed using 3-matic software for wrapping, smoothing, and mesh reduction to obtain a standardized pelvic 3D model (Fig. 2).

The fixation trajectory of the combined reduction and anatomical plate was drawn. The proximal end of the reduction plate started from the anterior inferior iliac spine, followed the posterior wall of the acetabulum, and extended to the distal end until a third of the way down the line between the greater ischial notch and the ischial spine, which was at the projection point on the fixation trajectory. It then curved to the quadrilateral body. The reduction plate and T-type auxiliary plate were fixed to the anterior column of the acetabulum. The locking plate began 1 cm below the anterior inferior iliac spine, parallel to the reduction plate, and extended distally to the apex of the ischial tuberosity (as shown in Fig. 1). The Trim command under the Finish function list in the 3-matic software was used to create the profile that crossed the centerline of the steel plate.

**Fig. 3:**
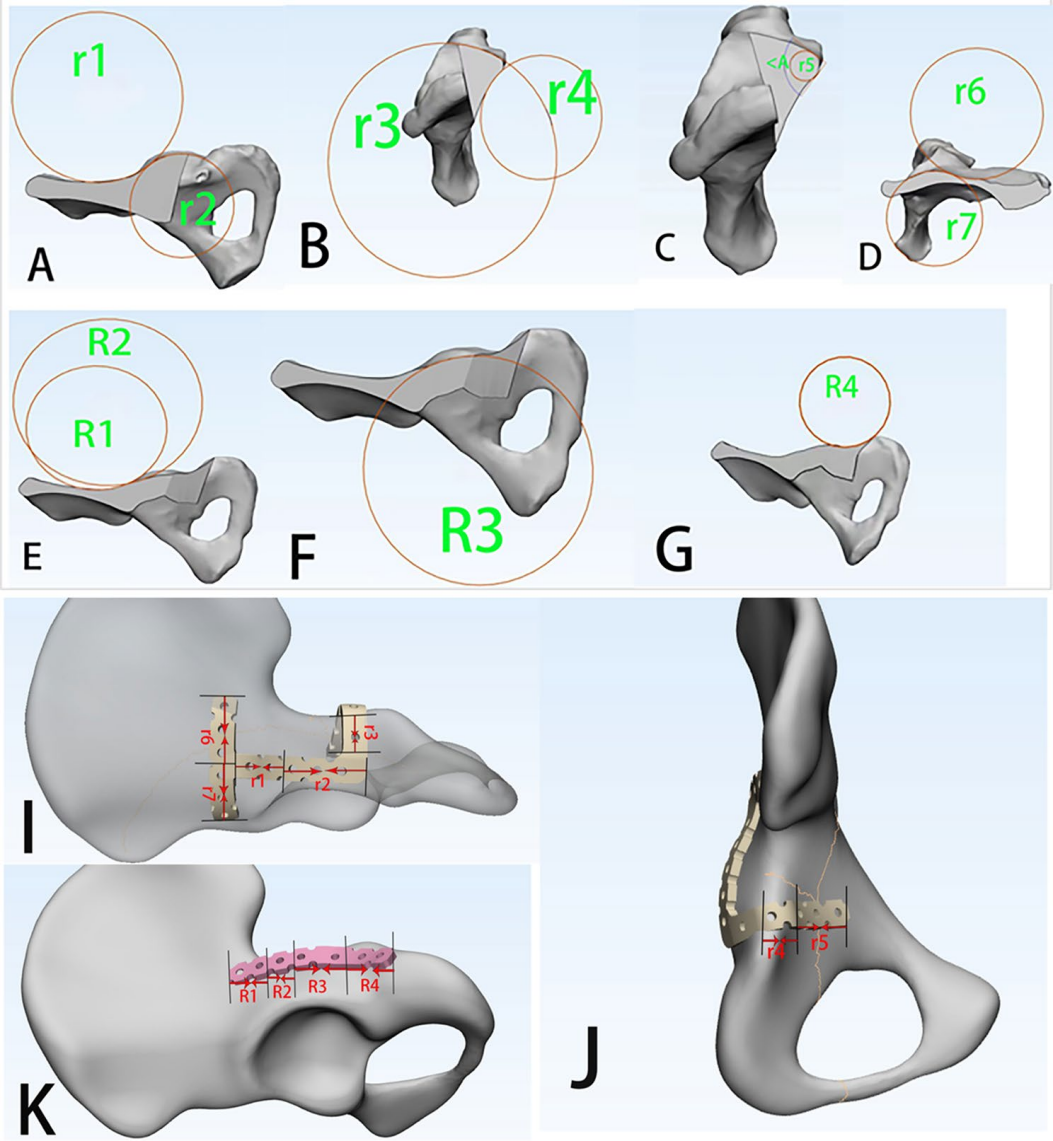
The fixed trajectory of the combined anatomical reduction plate was viewed as arcs with different radii of curvature. A to C are the five arcs of the reduction plate (r1, r2, r3, r4, and r5) and the bending angle (< A °). D is the two circular arcs of the T-type auxiliary plate (r6 and r7). E–G are the four circular arcs of the locking plate (R1, R2, R3, and R4). Each circular arc is fitted by the software, and the diameter and length of the circle can be measured directly by the software. The arc length of each circular arc can be calculated by the formula [20]: arc length = diameter × (arc in chord length/diameter). The length of the steel plate can be obtained by summing the arc lengths. Using the measuring tool in the software, the degree of bending of the reduction plate to the quadsquare was measured (< A °). I and J show the continuous irregular curve of the plate fixation trajectory

The fixation trajectory of the reduction plate can be considered as five arcs with the radii of curvature of the ilium (r1), posterior wall (r2), ischium (r3), quadrilateral area (r4), and bending area (r5). The radius of curvature of the locking plate can be divided into four arcs: the iliac region (R1), proximal posterior wall (R2), posterior wall region (R3), and distal posterior wall (R4). The fixed trajectory of the T-shaped auxiliary plate can be regarded as two arcs with radii of curvature at the posterior superior iliac spine side (r6) and the anterior superior iliac spine side (r7) (as shown in Fig. 3). Each arc was fitted using the software, and the diameter and length of the circle were measured directly using the software. The arc length of each arc can be calculated by the formula [20]: arc length = diameter × (arc in chord length/diameter), and the length of the plate was obtained by summing the arc lengths. Simultaneously, the degree of bending of the reduction plate to the square was measured (< A °) using the measuring tool in the software.

## Data analysis

SPSS 29.0 (IBM Corp., Armonk, NY, USA, Version 29.0) was used to process and analyze the data. All data were expressed as mean ± standard deviation (SD). Differences were considered statistically significant at *P* < 0.05.

## Results

### Comparison of the length of each section and the total length of the reduction plate between males and females

In this study, hemipelvic data were obtained from 51 males aged 22–60 years, with an average age of 44.37 years, The age difference was not statistically significant (*P* > 0.05). The average arc lengths of r1, r2, r3, r4, and r5 were 54.09 ± 5.30 mm (42.74–67.26 mm), 34.34 ± 4.57 mm (24.92–45.80 mm), 30.54 ± 2.99 mm (26.38–37.92 mm), 25.39 ± 4.11 mm (14.61–33.68 mm), and 7.77 ± 1.73 mm (4.52–13.82 mm), respectively, with a total length of 151.70 ± 11.55 mm (125.25–178.21 mm). In addition, hemipelvic data were obtained from 51 females aged 23–60 years, with an average age of 47.88 years, The age difference was not statistically significant (*P* > 0.05). The average arc lengths of r1, r2, r3, r4, and r5 were 53.80 ± 3.90 mm (47.01–68.78 mm), 32.48 ± 4.73 mm (23.98–44.93 mm), 26.97 ± 3.20 mm (21.25–34.30 mm), 22.39 ± 3.87 mm (13.15–31.17 mm), and 6.35 ± 1.70 mm (3.22–11.84 mm), respectively, with a total length of 141.99 ± 8.94 mm (117.56–158.19 mm). The statistical results indicate that there was no significant difference in the arc length of r1 between males and females (*P* > 0.05); however, the arc lengths of r2, r3, r4, and r5, and the total length of the reduction plate in females were significantly shorter than those in males (*P* < 0.05; Table 1).

**Table 1:** Comparison of the length of each zone and the total length of the reduction plate trajectory between males and females (mm, mean ± standard deviation)

Gender	*n*	Iliac (r1)	Posterior wall (r2)	Ischial (r3)	Quadrilateral (r4)	Bending zone (r5)	Total length
Male	51	54.095.30	34.34 ± 4.57	30.54 ± 2.99	25.39 ± 4.11	7.77 ± 1.73	151.70 ± 11.55
Female	51	53.80 ± 3.90	32.48 ± 4.73	26.97 ± 3.20	22.39 ± 3.87	6.35 ± 1.70	141.99 ± 8.94
t		0.308	2.026	5.834	3.798	4.178	4.747
P		0.759	0.045	< 0.001	< 0.001	< 0.001	< 0.001

### Comparison of length of each zone and total length of the locking plate between males and females

The average arc lengths of R1, R2, R3, and R4, and the total arc length in the 51 males were 36.61 ± 5.12 mm (24.86–47.66 mm), 14.06 ± 2.98 mm (7.80–21.37 mm), 32.62 ± 4.61 mm (19.50–43.78 mm), 32.18 ± 4.95 mm (21.33–45.19 mm), and 115.46 ± 8.82 mm (96.63–134.84 mm), respectively. The average arc lengths of R1, R2, R3, and R4, and the total arc length in the 51 females were 36.55 ± 5.18 mm (25.58–53.87 mm), 14.93 ± 3.43 mm (4.59–20.17 mm), 30.29 ± 4.46 mm (21.00–43.09 mm), 26.84 ± 4.44 mm (17.39–38.43 mm), and 108.61 ± 7.26 mm (93.70–123.13 mm), respectively.The statistical results indicate that there was no significant difference in the arc lengths of R1 and R2 between males and females (*P* > 0.05); however, the arc lengths and the total length of the locking plate for R3 and R4 in females were significantly shorter than those in males (*P* < 0.05; Table 2).

**Table 2:** Comparison of the length of each zone and the total length of the locking plate trajectory between males and females (mm, mean ± standard deviation)

Gender	*n*	Iliac (R1)	Proximal to posterior wall (R2)	Posterior wall (R3)	Distal to the posterior wall (R4)	Total length
Male	51	36.61 ± 5.12	14.06 ± 2.98	32.62 ± 4.61	32.18 ± 4.95	115.46 ± 8.82
Female	51	36.55 ± 5.18	14.93 ± 3.43	30.29 ± 4.46	26.84 ± 4.44	108.61 ± 7.26
t		0.053	1.366	2.592	5.729	4.286
P		0.958	0.175	0.011	< 0.001	< 0.001

### Comparison of the sectional length, total length, and bending angle (< A °) between males and females

The average values of the arc length, corresponding to r6 and r7, total length, and bending angle (< A °) in the 51 male pelvic T-shaped auxiliary steel plates were 28.16 ± 5.17 mm (18.42–42.31 mm), 46.50 ± 5.37 mm (33.97–62.22 mm),74.67 ± 6.81 mm (57.62–85.17 mm), and 83.58 ± 13.99° (56.63–114.79°), respectively. In the 51 female pelvic T-shaped auxiliary steel plates, the average values of the arc length, corresponding to r6 and r7, total length, and bending angle (< A °) were 27.09 ± 5.40 mm (15.28–39.83 mm), 42.61 ± 5.51 mm (23.94–53.96 mm),69.70 ± 6.82 mm (51.40–88.08 mm), and 71.99 ± 14.74° (42.06–104.71°). The statistical analysis indicated no significant difference (*P* > 0.05) in the arc length of r6 between males and females, while the arc length of r7 and the total length of the T-shaped auxiliary plate in females were significantly smaller than those in males (*P* < 0.05). The bending angle (< A °) in females was also significantly smaller than in males (*P* < 0.05; Table 3).

**Table 3:** Comparison of sectional length, total length, and bending angle (mm, °, mean ± standard deviation)

Gender	*n*	Posterior superior iliac spine side (r6)	Anterior superior iliac spine (r7)	Total length	Bending angle (< A °)
Male	51	28.16 ± 5.17	46.50 ± 5.37	74.67 ± 6.81	83.58 ± 13.99
Female	51	27.09 ± 5.40	42.61 ± 5.51	69.70 ± 6.82	71.99 ± 14.74
t		1.023	3.616	3.676	4.073
P		0.309	< 0.001	< 0.001	< 0.001

## Discussion

Digital three-dimensional reconstruction medical software is a very effective preoperative planning tool, especially in orthopedic surgery, which has been widely used and studied [21]. Among themMimics software is widely used to process and analyze medical images. Using Mimics software, the internal features of the pelvis can be observed arbitrarily and analyzed in detail on the computer. Zhang et al. [22] used the Mimics software to measure the safe range of quadrilateral screw placement; they made a clear definition of the “safe zone” and “dangerous zone” of screw placement, which greatly helped to quickly determine the optimal placement of screws during surgery. Guo et al. [23] used the Mimics software to measure and analyze the area and position of the thin cortical bone area of the quadrilateral surfaceand found that the area of the thin cortical bone area of the quadrilateral surfaceincreased with age. Therefore, special attention should be paid to changes in cortical bone thickness in young and old individuals when designing a fixation device for the quadratus body. Shang et al. [20] applied the editing, processing, measurement, and other functions of the Mimics software to measure the irregular trajectory of a steel plate, considering the trajectory of the steel plate as several circular arcs for segmental measurement. Using this measurement tool, the diameter and length of the circular arc can be measured, and the length of the circular arc can be calculated to design an anatomical steel plate that is more suitable for the pelvic anatomical structure.

The quadrangle is one of the important components of the acetabulum, which plays an important role in preventing femoral head displacement [20, 24]. Once a fracture of the quadrangular body occurs, it usually leads to hip dislocation, changes in the corresponding relationship between the joints, and mechanical imbalance. Poor reduction of the fracture with internal fixation during surgery leads to a reduction in the joint weight-bearing surface and stress concentration, which accelerates the degeneration of articular cartilage and the occurrence of traumatic arthritis. Therefore, quadrangular body fractures have always been a difficult problem in traumatic orthopedic treatments [24, 25]. 

In recent years, anatomical plates have seen significant progress, and many studies have been conducted on specific parts of the skeleton. To study and design anatomical plates, the anatomical morphology of the pelvic acetabulum is crucial. Many researchers have previously performed numerous measurements and studies on the anatomical morphology of the pelvic acetabulum. In a study of the acetabular posterior column anatomical plate conducted by Liu et al. [26]. , the fixed trajectory of the plate was regarded as three circular arcs of the posterior wall, proximal end, and distal end of the acetabulum, and the radius of curvature of the circular arc and the diameter of the femoral head were measured. The results showed no statistically significant difference in arc length between males and females, whereas the diameter of the femoral head was significantly smaller in females than in males. Wu et al. [27] measured and analyzed the width of the posterior acetabular column by using pelvic CT data; the results indicated that the width of the posterior acetabular column was smaller in females than in males, with a significant difference. Shang et al. [20] used digital software measurement technology to design a new dynamic anterior plate screw fixation system in line with the Chinese population. Wu et al. [28] used a new dynamic anterior plate-screw fixation system with a single ilioinguinal approach to treat complex acetabular fractures involving the quadrilateral body. results have demonstrated that use of this system is a safe and effective option for the treatment of complex acetabular fractures involving the quadrilateral surface .The results obtained by the digital software in this study were similar to those reported in the previous literature; therefore, the measurement method used in this study was reliable.

Currently, the most widely used internal fixator for complex acetabular fractures is a flexible reconstruction plate that facilitates intraoperative shaping. However, owing to the complex fracture types being diverse and irregular, an imprecisely shaped plate will lead to unstable fixation [1]. For inexperienced doctors, the accuracy of plate shaping according to the morphology and anatomical structure of the fracture is poor, which may lead to loss of reduction after surgery. Repeated shaping of the plate during the operation also easily causes the plate to break, increases the operation time and bleeding, and increases the risk of infection [29, 30]. In addition, for complex acetabular fractures, such as anterior column posterior hemitransverse fractures, double-column fractures, and T-type fractures, combined approaches are often required for reduction and internal fixation; however, combined approaches have the disadvantages of longer operation time, more blood loss, and greater surgical trauma [31, 32]. 

Therefore, it is necessary to improve the existing anatomical plates to reduce the difficulty of surgery and obtain good results. Based on the current situation, we innovatively designed the combined reduction anatomical plate. This fixation system consists of two parts: a locking plate and a reduction plate. The locking plate was an arc-shaped strip plate fitted above the greater sciatic notch. The reduction plate was bent to fit the anatomical structure above the acetabulum and the quadrilateral body. It is mainly used for the treatment of complex acetabular fractures involving the quadrangular body (such as T-type, anterior column and posterior hemitransverse, and both-column fractures). The fixation method was obtained from a Chinese National Patent (patent no. CN202222137846.2).

The fixed trajectory of this system was regarded as several continuous irregular curves, and the best circular arc was fitted to each curve using the software. The diameter and length of the circle can be measured directly using software, and the arc length of each circular arc can be calculated using the conversion formula: [20] arc length = diameter × (arc in chord length/diameter). The arc lengths are then summed to obtain the final plate lengths. Since the arc determines the shape of the plate, studying the arc is crucial for designing steel plates. To ensure consistency in the positions of all the plate models, the same marker points were selected for measurement. This device uses CT data to reconstruct a 3D model of the pelvis, and the fixed trajectory of the steel plate was measured. It is an anatomical plate with high accurac, which has the advantages of reducing the time required for shaping the steel plate during the operation and reducing the risk of surgery. This fixation device is mainly used for the treatment of quadrilateral acetabular fractures (such as T-type, anterior column and posterior hemitransverse, and both column fractures) and has a better reduction and blocking effect on quadrilateral body fractures combined with central dislocation of the hip. While the posterior column was fixed, the T-type auxiliary plate also had good reduction and fixation effects when combined with the anterior column.

**Fig. 4:**
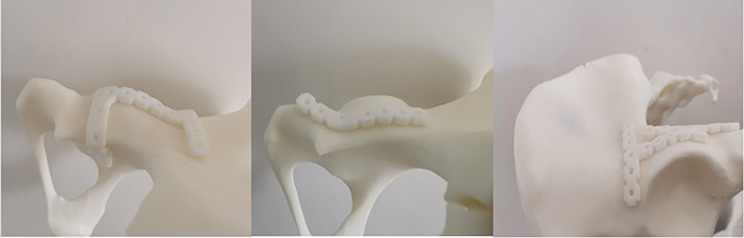
The combined anatomical reduction plates selected for the digital measurement in this study has high accuracy

Generally, the skeletal morphology of the human body varies greatly depending on sex, age, geographical location, and population. Usually, a small error is acceptable and does not significantly affect the results. Choosing a reduction plate and locking plate with the wrong curvature to treat fractures may lead to postoperative reduction loss and complications such as traumatic arthritis; therefore, it is necessary to select an appropriate plate during the operation (as shown in Fig. 4).

The combined reduction anatomical plate in this study is an anatomical plate, it accuracy was higher than that of traditional reconstruction plates and can significantly reduce the difficulty of shaping the plate during operation. However, this study has some limitations. For example, our measurements were representative of specific populations and may not be generalizable to all races. Typically, pelvic anatomy varies greatly depending on the geographical location and population. Previous studies have shown a decreasing trend in pelvic size from high to low latitudes [33]. In addition, the different physiological functions of men and women determine morphological differences in the pelvis [34]. In the software measurement process, errors caused by human factors are inevitable when the landmarks are determined. Additionally, screws were not considered in this study, and in a follow-up study, we will further optimize them to improve the treatment effect.

## Conclusions

In this study, digital technology was used to measure the fixation trajectory of a combined anatomical reduction plate, which provided an anatomical basis for plate design. Simultaneously, it overcomes the shortcomings of traditional cadaveric bone samples and large measurement errors. Although the anatomical plate we designed could not meet the anatomical structure of all populations, its accuracy was higher than that of traditional reconstruction plates. It can be used to treat quadrilateral acetabular fracture. so that the treatment methods become diversified.Finally, the clinical outcomes of the fixation devices used in this study will be investigated in future studies.

## Data Availability

The datas used and analyzed during the current study are available from the corresponding author on reasonable request.
